# Boston Dental College

**Published:** 1882-05

**Authors:** 


### BOSTON DENTAL COLLEGE.

The fourteenth annual commencement.

The number of matriculates for the session was fifty.

The degree of D.D.S. was conferred on the following graduates by the President of the College, Professor Isaac J. Wetherbee.

| NAME. | RESIDENCE. |
|---|---|
| Rufus Oscar Clark | Massachusetts. |
| Frank Arthur Damon | Massachusetts. |
| Harry Neal Day | Massachusetts. |
| Homer Emerson | Massachusetts. |
| Edwin Chas. Frizzell | Vermont. |
| Charles Sumner Gates | Massachusetts. |
| Edw'd Payson George | New Hampshire |
| Wm. Franklin Gilman | Massachusetts. |
| Alfred Henry Gilson | Massachusetts. |
| Wm. Osceola Gray | Massachusetts. |
| Charles W. Ham | NewHampshire |
| Newell D. Johnson | Massachusetts. |
| John Francis Lennon | Massachusetts. |
| George Otis Mitchell | Maine. |
| Asa Stevens Nutter | Canada. |
| Walt. Leonard Stevens | Massachusetts. |
| Byron Howard Strout | Alabama. |
| Theron W. Temple | Massachusetts. |
| Augustus L. Wells, Jr | Rhode Island. |

